# Qualitative Study on Vaccinations for Travelers

**DOI:** 10.3390/vaccines14010047

**Published:** 2025-12-30

**Authors:** Fabiana Nuccetelli, Sara Ciampini, Valeria Gabellone, Patrizio Zanobini, Pierluigi Lopalco, Luigi Roberto Biasio

**Affiliations:** 1 Department of Experimental Medicine, University of Salento, 73100 Lecce, Italy; fabiana.nuccetelli@unisalento.it (F.N.); valeria.gabellone@unisalento.it (V.G.); pierluigi.lopalco@unisalento.it (P.L.); 2 UOC—Vaccination and International Centre Vaccination, ASL Roma 1, 00193 Rome, Italy; sara.ciampini@aslroma1.it; 3 Department of Health Sciences, University of Florence, 50134 Florence, Italy; patrizio.zanobini@unifi.it; 4 Scientific Committee Giovanni Lorenzini Foundation, Viale Piave 35, 20129 Milan, Italy

**Keywords:** vaccine hesitancy, travel vaccinations, health communication, vaccination behavior, 3Cs model

## Abstract

**Background:** Vaccinations are essential to protect travelers from infectious diseases, especially in high-risk destinations. However, awareness and adherence to vaccination recommendations vary, influenced by communication, personal beliefs, and behavior. **Methods:** A focus group was conducted in February 2025 at a local health authority in central Italy, specifically within its travel clinic, to explore travelers’ awareness, attitudes, and behaviors regarding vaccination. The discussion was analyzed using the “3Cs” Vaccine Hesitancy model. Participants were purposively selected to ensure diversity and representativeness. Discussions included past travel experiences, knowledge of required vaccines, motivations for immunization, and barriers to access. **Results:** Four key thematic areas emerged: socio-cultural/environmental factors, psychological/emotional influences, knowledge/information access, and general health perceptions. Communication gaps often weakened belief in vaccine efficacy and necessity. Cultural background, past experiences, and risk perception heavily influenced decisions. **Discussion:** Although vaccination is widely viewed as a protective measure, vaccine hesitancy persists due to misinformation and limited institutional trust. The COVID-19 pandemic intensified both awareness and skepticism. The 3Cs model clarified hesitancy levels and barriers, emphasizing the need for effective communication and trust-building. **Conclusions:** Enhancing access to accurate information, strengthening healthcare professionals’ communicative role, and reducing economic obstacles are crucial. Tailored awareness campaigns and integrated health policies are essential to increasing vaccine uptake, safeguarding traveler health, and limiting global disease spread. **Patient or Public Contribution:** Members of the public contributed to this study by participating in a focus group, where they shared their personal experiences, perceptions, and opinions regarding travel-related vaccinations. Their insights provided valuable qualitative data that helped inform the study’s findings. However, they were not involved in the study design, the analysis of the data, or the preparation of the manuscript. The role of participants was limited to the data collection phase of the study.

## 1. Introduction

Vaccinations are a fundamental tool for the prevention of infectious diseases, particularly for travelers heading to destinations at risk of exposure to emerging or endemic diseases. The World Health Organization (WHO) emphasizes that vaccination is a cornerstone of global public health, capable of preventing the spread of cross-border infectious diseases and reducing health risks for travelers [1]. These aspects are also exacerbated by the impact of climate change, which influences the distribution of infectious diseases, and the increasing spread of diseases in areas previously considered low-risk [2], at a time of the remarkable rebound recovery of international travel [3]. In this context, improving communication and access to health information is crucial to optimize public health protection and prevent the global spread of diseases.

According to the current Italian National Vaccination Prevention Plan [4], pre-travel consultation is fundamental to identify risk factors, to define behavioral guidelines, vaccination schedules, and pharmacological prophylaxis; it helps the informed traveler understand the risks associated with travel; and promotes individual empowerment against often underestimated diseases. It is therefore an opportunity to describe and discuss the risks and develop plans that minimize these risks. Necessary vaccinations must be personalized based on the individual traveler’s vaccination history, travel itinerary, amount of time available before departure, and the individual’s degree of fragility and susceptibility.

However, despite clear guidelines from health authorities and the availability of effective vaccines, travelers show inconsistent awareness of and adherence to vaccination recommendations, with significant variations depending on destination, cultural background, information sources, and individual perceptions of vaccine safety and efficacy. In particular, pre-travel health preparation is influenced by several factors, including knowledge of prevalent diseases and outbreaks in the destination regions, access to official information, and personal and social perceptions of the need for vaccination.

Recent studies show that although travelers have some awareness of the importance of vaccinations, they often lack detailed and up-to-date knowledge about those specifically required or recommended for each type of travel [5]. This is further complicated by growing misinformation regarding vaccine safety, which can negatively affect individual choices [6]. Additionally, while the phenomenon of vaccine hesitancy is not new [7], it resurged prominently during the COVID-19 pandemic [8,9].

This exploratory study—promoted by the Department of Health Sciences at the University of Florence and the Department of Experimental Medicine at the University of Salento—aimed to identify psychological antecedents of travel vaccine hesitancy through focus group methodology. Rather than seeking exhaustive understanding across demographic subgroups (meaning saturation), our objective was to capture the breadth of themes and barriers present in individuals seeking pre-travel medical advice (code saturation) to also inform future research.

## 2. Methods

This qualitative study explored various aspects, including past travel experiences, sources of information about required vaccinations, motivations driving vaccination decisions, and obstacles hindering access to vaccinations. The decision to analyze these factors was considered essential for understanding the dynamics that shape vaccination choices and for developing more effective strategies to promote vaccinations among travelers.

The study was conducted through a focus group, moderated by a psychology healthcare expert. Participants were selected according to criteria of heterogeneity to ensure diverse representation in socio-demographic characteristics and travel experience. Eligible participants were adults (≥18 years) scheduled to receive travel-related vaccinations at the Vaccination Center of ASL Roma 1 (Rome, Italy) during the study period. Given the clinic’s status as one of Italy’s largest international vaccination clinics with a diverse traveler population, this single-site sampling approach aligns with the exploratory objective of identifying psychological antecedents of vaccination behavior, rather than achieving comprehensive demographic representation across stratified subgroups.

Inclusion criteria were: (i) upcoming international travel to destinations for which vaccinations are recommended or mandatory; (ii) ability to understand and communicate in Italian; and (iii) willingness to provide written informed consent. Exclusion criteria included age under 18 years and inability to understand or sign the informed consent form.

A few days prior to the scheduled vaccination session, individuals booked for vaccination were contacted by telephone by trained clinic staff, following the chronological order of appointments. Potential participants were informed about the study aims and procedures and invited to voluntarily participate in a focus group discussion lasting approximately one hour. Recruitment continued until code saturation was achieved, ensuring diversity in gender, age, and educational background, thereby enhancing the credibility and transferability of the qualitative findings.

**Figure 1:**
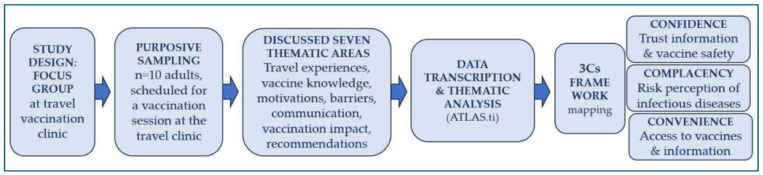
Qualitative study design flowchart. Purposive sampling (n = 10) recruited participants for a moderated focus group exploring seven thematic areas: travel experiences, vaccination knowledge, motivations, barriers, communication, vaccination impact, and recommendations. Data were transcribed and analyzed using thematic analysis (ATLAS.ti) to identify emerging themes, which were then mapped onto the Vaccine Hesitancy 3Cs framework—Confidence (trust in vaccine safety), Complacency (risk perception), and Convenience (vaccine accessibility)—to identify psychological antecedents of vaccination behavior and inform communication strategies for improving vaccination adherence among travelers.

The total number of participants was 10, with 6 females, and the average age was 45 years (range 27–60, median 47.5). All participants were of Italian nationality. Regarding education level, all participants had at least a high school diploma, with some holding university degrees. The session was recorded with respect to privacy, then transcribed and analyzed using thematic analysis techniques [10]. The transcripts were categorized using ATLAS.ti ver 22 software for in-depth analysis of the qualitative data, dividing responses into specific codes and then grouping them into broader thematic categories [11] (Figure 1).

The study was authorized by the Ethics Committee of the University of Florence (n. 359 of 27 November 2024). The focus group was conducted in February 2025; exact dates are not reported to protect participant privacy. Participation was subject to obtaining written informed consent from all participants before entering the focus group.

The focus group questions explored the following thematic areas:

1. Previous Travel Experiences
  ○ Destinations visited and motivations for choosing them.
  ○ Sources of information regarding necessary vaccinations.
2. General Knowledge about Vaccinations
  ○ Perceived importance of vaccinations for international travel.
  ○ Knowledge of recommended and mandatory vaccinations.
  ○ Access to information from official entities [12].
3. Motivations for Vaccination
  ○ Factors influencing the decision to get vaccinated.
  ○ Sources of information considered most reliable.
  ○ Opinions on mandatory vaccinations for entry into certain countries.
4. Barriers and Uncertainties
  ○ Obstacles to accessing vaccinations.
  ○ Perception of the safety and effectiveness of vaccines [13].
  ○ Influence of cost on vaccination decisions.
5. Communication and Advice
  ○ Ease of obtaining information about vaccinations.
  ○ Experiences with healthcare professionals and official sources.
  ○ Strategies to improve communication in the healthcare field.
6. Impact of Vaccinations on Health
  ○ Experiences of diseases contracted while travelling that could have been prevented by vaccination.
  ○ Perception of the effectiveness of vaccinations in protecting health.
7. Conclusions and Recommendations
  ○ Importance of vaccinations in the health prevention of travelers.
  ○ Suggestions for improving adherence to vaccination recommendations.

### 2.1. Integration of the 3Cs Model

The vaccine hesitancy “3Cs” Model (Confidence, Complacency, Convenience) [14] was used to analyze the participants’ responses and explain the key psychological factors influencing attitudes and behaviors towards vaccination (also defined as psychological antecedents of vaccination). Although other models have been proposed concerning components of vaccine hesitancy [9,15], the 3Cs model remains a schematically useful interpretative framework for understanding the psychological factors influencing people’s attitudes, such as those towards vaccination (vaccine acceptance) and intention to vaccinate (vaccine intention), considered a precursor to behaviors, like actual vaccine uptake. Applying this model allows for a better understanding of the barriers and facilitators to vaccination among travelers, providing insights for more effective communication strategies and public health promotion.

- Confidence: Confidence in the information provided and the safety of vaccines. It was explored how travelers who rely on official sources (e.g., healthcare professionals and government bodies) tend to have more positive attitudes towards vaccination.
- Complacency: The perception of the risk associated with infectious diseases. The tendency of some travelers to underestimate the risk of contracting diseases, especially in destinations considered low-risk, was investigated.
- Convenience: The ease of access to vaccinations and health information. Factors related to the availability and accessibility of information, including healthcare advice and digital platforms, were explored to facilitate the adoption of vaccination behaviors.

### 2.2. Statistical Analysis

In line with emerging qualitative methods, we conducted quasi-statistical analyses of coded qualitative data to assess potential biases, using numerical summaries to sustain validity while preserving core qualitative insights. Post hoc analyses at a 95% confidence level characterized response homogeneity/heterogeneity and explored groupthink and social desirability as potential bias sources. These analyses included internal consistency, dimension-specific variance, bivariate correlations, age and sex stratification, and the distribution of positive versus negative responses among travelers, as classified by ATLAS.ti. Given the limited sample size, exact tests with 1.000 permutations (Kruskal–Wallis and chi-squared) were employed to ensure adequate power. These analyses were not pre-specified and should be interpreted as descriptive rather than inferential. Statistical software used: Jamovi v2.7.6.0 and Stata v17.0.

## 3. Results

The qualitative data collected through the focus groups, analyzed using ATLAS.ti software, allowed the transcription to be divided into 161 codes and 137 categories, which were subsequently grouped into four main macro-categories. These macro-categories were further divided into specific sub-categories that reflect the dynamics influencing travelers’ vaccination choices, aligned with the 3Cs framework (Table 1). Below, the main results are presented according to the three components of the model:

### 3.1. Confidence

Confidence in vaccination-related information was a central theme in the focus groups, with participants expressing a clear relationship between trust in official sources and adherence to vaccination recommendations.

○ Socio-cultural adaptation: Participants tended to trust the information received from travel agencies or doctors without conducting independent research, but this happened primarily when the source was considered reliable. This trust in institutional sources facilitated their vaccination preparation.
▪ Example quote: *“I booked the trip through an agency that told me there would be no issues with vaccinations. I didn’t worry too much.”* (Dialogue 1)
○ Emotions: Emotions played a crucial role in strengthening confidence in vaccinations. Participants felt calmer and safer after being vaccinated, seeing vaccination as a way to reduce anxiety.
▪ Example quote: *“When I had the vaccinations for the trip, I felt more relaxed. Now I’m not afraid of getting sick because I know I’m protected.”* (Dialogue 4)

### 3.2. Complacency

The perception of risk and the general attitude towards vaccination were strongly influenced by the concept of complacency, with participants tending to underestimate the risk, especially for destinations considered low-risk.

○ Travel: Vaccination was perceived as less necessary for trips to European destinations, which is a clear sign of complacency regarding the risk of diseases in these areas.
▪ Example quote: *“For Hungary, I didn’t worry about getting vaccinated. There was nothing that made me think it was necessary.”* (Dialogue 3)
○ Lack of information: Insufficient accurate information contributed to risk of misperception and reduced motivation to get vaccinated. Many participants reported difficulties finding clear and timely information on required vaccinations, contributing to complacency.
▪ Example quote: *“I never know who to turn to for detailed information on vaccines. Neither my doctor nor the pharmacy gives me clear answers.”* (Dialogue 7)

### 3.3. Convenience

The ease of access to vaccinations and health information was essential in promoting adherence to vaccination recommendations. The accessibility of information was identified as one of the main factors influencing vaccination decisions.

○ Empowerment: Participants who took the initiative to independently research information, through sources such as the Internet, expressed a greater sense of control over their health and a stronger inclination to get vaccinated.
▪ Example quote: *“Before I leave, I always check online to see what type of vaccine I need. I don’t completely trust the information I get only from my doctor.”* (Dialogue 8)
○ Healthcare & COVID-19: The COVID-19 pandemic highlighted the importance of convenience in accessing vaccinations. Growing mistrust of vaccines and increased resistance to vaccination were exacerbated by difficulties accessing clear information during the health crisis.
▪ Example quote: *“Before the pandemic, I didn’t question vaccines much. Now, I question everything.”*

### 3.4. Other Factors

In addition to the factors related to the 3Cs, aspects related to overall well-being and health during travel also emerged.

○ Holistic development: Vaccination is seen as part of a comprehensive well-being strategy while travelling. Participants often consider vaccination as one of many necessary precautions, alongside other preventive measures.
○ *Example sentence: “Vaccination is important, but it is just one part of how I take care of my health when travelling. I also make sure to take digestive medicine because I know my body can react differently in other countries.”* (Dialogue 10)

## 4. Discussion

The results of the qualitative analysis show how the psychological antecedents of vaccination influence travelers’ vaccination decisions. For example, confidence in official sources and clear information are decisive factors in vaccination adoption, while complacency related to risk perception and convenience in accessing information and vaccination facilities are factors that either hinder or facilitate vaccination behavior. The COVID-19 pandemic significantly affected complacency and trust, influencing perceptions about vaccine safety. Implementing strategies that enhance trust, reduce complacency, and simplify access to vaccinations would be essential for improving global public health.

The analysis of these attitudinal dimensions provides valuable insights for developing intervention strategies: improving trust through clear, evidence-based communication, increasing risk perception where complacency is high, and simplifying access to vaccinations and health information. Such strategies, if implemented in an integrated manner, could contribute to strengthening public health protection, reducing misinformation, and promoting greater empowerment for travelers in managing their health. Vaccinations indeed represent one of the most effective preventive tools against infectious diseases, especially for travelers heading to destinations with high risk of exposure to emerging or endemic diseases.

Our interview analysis identified key factors influencing vaccination perception and adoption among travelers, organized within three dimensions of the psychological antecedents framework: confidence, complacency, and convenience.

**Socio-cultural and Environmental Factors**—Were primarily associated with the confidence and complacency dimensions.

Socio-cultural adaptation

The research revealed that many people adapt to the vaccination regulations of their destination countries mainly in a reactive way, relying on information provided by third parties, such as travel agencies, doctors, or local health authorities. As one interviewee stated: *“When we travel, we usually rely on the information provided by travel agencies. We don’t have the time to search for all the information ourselves.”* This behavior reflects a form of “passive adaptation”, where individuals seek simple and immediate solutions rather than conducting independent research. These findings align with our earlier observations on socio-cultural adaptation, highlighting how passive reliance on external sources may limit travelers’ ability to make informed vaccination decisions. The literature supports this observation, indicating that many individuals rely on external sources rather than undertaking personal research to make health decisions [16].

Several travelers displayed low vaccine literacy levels, often deferring to travel agencies or healthcare providers rather than navigating complex vaccination requirements independently. This passive approach may undermine autonomy essential for sustainable vaccination uptake.

Diversity

Cultural differences significantly influence vaccination perceptions and practices. In many cases, travelers from countries with strong traditions of alternative medicine or different approaches to health may not perceive vaccines with the same urgency as those from countries with healthcare systems strongly oriented towards prevention. One interviewee said: *“In my country, we are very focused on traditional medicine, so we don’t see vaccines as a primary solution.”* Cultural diversity can indeed lead to different approaches to vaccination, with significant implications for international health policies [17].

Travel

For many participants, vaccination is seen more as an administrative formality than a health necessity. One interviewee remarked: *“Since I needed it for the visa, I got it, but I didn’t think it was that important for my health.”* This phenomenon is reflected in the literature, where vaccination is sometimes described as an ‘administrative barrier’ rather than a preventive health measure [18]. However, some travelers, particularly those heading to high-risk health countries, view vaccination as essential for their protection.

**Psychological and Emotional Factors**—Were Influenced by Confidence

Emotions

Emotions, such as the fear of contracting infectious diseases and trust in vaccines, play a significant role in determining attitudes towards vaccination. One interviewee said: *“The fear of contracting diseases while travelling is strong, so I get vaccinated to feel safer.”* Other participants expressed confidence in vaccines as a protective measure. The literature confirms that fear and trust are key determinants in health-related decisions [19].

Mental Health

The link between vaccination and psychological well-being was noted by many participants, several of whom indicated that vaccination reduced their anxiety regarding health during travel. One participant said: *“When I’m vaccinated, I feel more at ease during the trip. It helps reduce my health anxiety.”* According to Cohen et al. (2007), having control over one’s health is correlated with greater psychological well-being [20].

Positive Mindset

A positive attitude towards vaccination facilitates the adoption of preventive behaviors. For example, one participant stated: *“I always try to have a positive attitude towards health. Vaccination is a preventive measure that helps us protect ourselves before we leave.”* The literature confirms that people with a positive attitude towards health and prevention are more likely to engage in protective behaviors [21].

**Knowledge and Access to Information**—Were Driven by Convenience

Lack of Information

A recurring issue reported by many interviewees was the difficulty in accessing clear and accurate information regarding vaccinations. As one interviewee said: *“I’ve never found clear information about which vaccines I should get for my trip.”* Misinformation or a lack of appropriate information is a significant barrier to prevention [22]. Many participants reported difficulties in finding reliable guidance on the vaccinations required for their travel destinations. This emphasizes the barrier posed by inadequate access to official information, reinforcing the importance of improving communication channels to support informed vaccination choice.

Healthcare & COVID-19

The impact of the COVID-19 pandemic on vaccination perceptions was clearly evident in the interviews. Many participants noted an increase in awareness about vaccination, but also a growing skepticism and distrust towards health institutions. As one interviewee said: *“The pandemic made me reflect a lot on the importance of vaccination, but at the same time, it fueled some fears.”* This phenomenon has been widely documented in the literature [23]. The COVID-19 pandemic had a significant impact on vaccination perceptions, increasing both an awareness of the importance of prevention and, on the other hand, the level of skepticism towards health institutions. These results illustrate the dual effect of the COVID-19 pandemic, which not only raised awareness of the importance of vaccination but also fueled mistrust towards health institutions.

**Holistic Development**

The global approach to health, which views vaccination as part of a broader system of prevention and well-being, was highlighted by many participants. For example, one interviewee stated: “*Vaccination is important, but I believe health is a combination of things: healthy eating, physical exercise, and prevention.*” This perspective reflects a systems-based understanding of health that aligns with modern conceptions integrating physical, mental, and social aspects [24]. Within this holistic framework, vaccination serves as a foundational protective element that enables—rather than replaces—other health-promoting behaviors. Vaccination decisions are thus embedded within travelers’ broader health strategies and lifestyle choices, where immunization works synergistically with nutrition, exercise, hygiene, and preventive medications.

Within the context of travel medicine, vaccination should be understood as a core component of a broader holistic health development framework, an integrated approach to travelers’ health that encompasses clinical protection (vaccinations and pharmaceutical prophylaxis), behavioral prevention (safe food and water practices), psychological preparedness, risk awareness, and access to reliable health information. Findings from this study indicate that travelers often perceive vaccination not merely as a biomedical intervention, but as part of a comprehensive strategy to maintain well-being, reduce anxiety related to health risks, and increase confidence during travel. When embedded within structured pre-travel counseling, vaccination may act as a catalyst for enhanced vaccine literacy and personal empowerment, encouraging the adoption of preventive behaviors beyond immunization alone.

This integrated perspective aligns with contemporary public health approaches that emphasize prevention, informed decision-making, and continuity of care. Strengthening vaccination communication within a holistic preventive framework may therefore improve adherence to recommendations, mitigate vaccine hesitancy, and support travelers in developing sustainable, health-conscious behaviors before, during, and after travel. Understanding vaccination within this comprehensive context has important implications for health communication, as messaging that connects vaccination to travelers’ existing health practices and personal health philosophies may be more persuasive than disease-focused messages alone.

As mentioned, one of the key findings was the tendency toward passive adaptation to vaccination regulations, characterized by a reliance on external sources without independent research. This pattern reflects inadequate levels of interactive and critical vaccine literacy entailing not only knowledge and skills, but also motivation to seek, understand, and apply vaccination information [25]. Vaccine literacy is conceptualized as multidimensional, encompassing not only factual knowledge but also the ability to critically evaluate information and make autonomous health decisions. Our findings suggest that several travelers lack the interactive literacy skills necessary to navigate complex vaccination requirements independently, instead deferring to travel agencies or healthcare providers. This passive approach may undermine the autonomy essential for sustainable vaccination uptake. Previous research using online surveys [26,27] has demonstrated negative correlations between vaccine literacy levels and the 3Cs. Enhancing vaccine literacy—particularly interactive and critical dimensions—could complement confidence-building strategies by enabling travelers to evaluate information independently, distinguish reliable sources, and understand the rationale behind vaccination recommendations. Such an approach would address both the informational barriers and the motivation deficits we observed.

## 5. Limitations

### 5.1. Sample Composition and Representativeness

Our study had several limitations, most inherent to the qualitative nature of survey research. The 10-participant group is comparable to other vaccination communication studies [28], and its size falls within the WHO-recommended range of 5–12 participants per focus group regarding vaccines [29]. The study achieved code saturation appropriate for exploratory research [30], successfully identifying four distinct thematic categories that captured the range of issues rather than pursuing meaning saturation across demographic subgroups. Although the focus group included participants with variation in gender and age, all participants were united by actively seeking vaccination services. Nevertheless, the small sample size (n = 10) limits the generalizability of the results, particularly regarding the representativeness of traveler populations across different geographical regions and demographic profiles.

While participants demonstrated heterogeneity in selection, they may not have been truly representative of the target population of travelers. The sample included a high number of highly educated individuals. Educational imbalance is inherent to qualitative research that demands substantial time and effort, as resulting samples tend to skew towards individuals who feature greater openness, knowledge, and interest in the research [31]. However, the participants’ demographic profile—predominantly advanced degree holders with an average age of 45 years—aligns with international traveler characteristics, consistent with Italian national statistics [32].

Although selection bias may exist, our sample was directly relevant for public health intervention, as it represents the intended user population—travelers attending vaccination clinics where counseling must address vaccinee attitudes and concerns to optimize travel protection. To strengthen future research, we recommend recruiting larger participant samples with varied educational backgrounds, socioeconomic statuses, and ethnic profiles across multiple travel clinics in different geographical contexts. This would characterize vaccine literacy and hesitancy profiles across diverse populations and enable the validation of thematic findings through quantitative approaches.

### 5.2. Social Desirability and Groupthink

Focus groups carry inherent risks of social desirability and groupthink bias [10]. However, the research literature shows that quasi-statistics derived from qualitative data can strengthen validity assessments by identifying patterned responses and discrepant cases [33]. Triangulating multiple data sources reduces—though does not eliminate—these limitations [34]. In focus-group analysis, social desirability and groupthink are relatively straightforward to detect with quasi-quantitative methods as they produce observable, patterned responses across participants.

We conducted multi-method statistical testing to detect potential social desirability:

- Cronbach’s alpha indicated excellent internal consistency for overall positive responses (α = 0.906); alpha remained high after removing each dimension (range α = 0.855–0.898), suggesting the scale was reliable and not driven by any single dimension.
- Response distribution analysis: the proportion of positive responses showed substantial heterogeneity (range 0–1.0, mean = 0.42 ± 0.40), with participants stratifying into three distinct groups: 30% expressed entirely negative responses, 40% showed mixed responses, and 30% predominantly positive responses. This wide distribution contradicts the clustering toward socially favorable endpoints expected under social desirability bias.
- Kruskal–Wallis permutation testing revealed no significant association between individual travelers and positive response counts (χ^2^ = 8.727, *p* = 1.0000).
- Chi-square tests for independence confirmed no relationship between traveler identity and positive responses (χ^2^ = 60.0, *p* = 0.267) or negative/neutral responses (χ^2^ = 50.0, *p* = 0.281), indicating that response patterns were random rather than systematically biased toward favorable or negative endpoints.

These convergent findings—high internal consistency, wide response distribution, absence of individual dominance, and random distribution across positive and negative responses—provide evidence against social desirability bias. No individual or subgroup systematically elevated positive responses to appear more favorable.

We also conducted variance analysis across thematic dimensions to assess potential groupthink:

- Responses revealed variation across domains (socio-cultural dimension variance = 0.62 ± 0.79; psychological = 0.18 ± 0.42; information access = 1.16 ± 1.07; well-being = 0.54 ± 0.74). The limited psychological dimension variance (0.18) could reflect groupthink, whereby individuals may conceal their emotional expressions in group settings [35]. However, the substantially higher variance in the information access dimension (1.16) indicates that homogeneity was not uniformly distributed across all domains, suggesting dimension-specific rather than global groupthink.
- Modest age-related variation was observed in psychological responses, with younger participants scoring 2.0 while middle-aged groups ranged from 1.67–1.75, suggesting responses were not entirely uniform across demographic subgroups.
- Kruskal–Wallis permutation testing found no significant association between individuals and overall response patterns (χ^2^ = 8.782, *p* = 1.0000), indicating no single participant or subgroup monopolized the discussion.
- Substantive response diversity: response counts across dimensions ranged widely (mean positive responses 4.40 ± 4.33; mean negative responses 4.30 ± 2.91), indicating disagreement on substantive issues.

Combined findings—dimension heterogeneity, demographic variation, absence of individual dominance, and response diversity—counter a groupthink interpretation and suggest psychological convergence may reflect genuine emotional consensus. To strengthen validation of emotional dimensions in future research, we recommend complementary methods such as anonymous surveys or individual interviews.

## 6. Future Research

In medical research, qualitative methods are useful to generate hypotheses and understand mechanisms, while quantitative methods are superior for testing hypotheses and establishing prevalence. The most robust approach often combines both—using qualitative work to inform survey design and validate quantitative findings. Our exploratory focus group findings will be complemented by an ongoing quantitative study—online survey—to confirm significant associations between higher vaccine literacy, and lower hesitancy among travelers. Notably, vaccine hesitancy remains understudied in this population [9]. To strengthen future research, we recommend recruiting larger participant samples with varied educational backgrounds, socioeconomic statuses, and ethnic profiles across multiple travel clinics in different geographical contexts. This would better characterize vaccine literacy and hesitancy profiles across diverse populations and enable the validation of our thematic findings through quantitative approaches.

## 7. Conclusions

The study highlighted how travelers’ vaccination decisions are shaped by socio-cultural, psychological, informational, and health-related factors. The 3Cs model revealed that emotions, trust in health institutions, and access to information are central to vaccination uptake.

These findings underscore the need for effective communication strategies that strengthen institutional trust, improve access to clear information, and empower travelers’ health decisions. The study also suggests integrating vaccination within a broader preventive health framework—encompassing diet, hygiene, and other preventive measures—to encourage overall well-being in travelers.

Further research should examine relationships between travelers’ attitudes, socio-demographic backgrounds, vaccine literacy levels, and the influence of social media and awareness campaigns on perceptions and behaviors through qualitative, quantitative, and implementation studies. Enhancing vaccine literacy, particularly its interactive and critical dimensions, could support informed, autonomous, and sustainable vaccination uptake.

## Figures and Tables

**Table 1:** Summary of key findings from the focus groups, divided according to the psychological antecedents of vaccination (the ‘3Cs’ model).

Model Factor	Key Themes	Summary of Findings
Confidence	Trust in official and institutional sources	Participants primarily rely on official sources (such as travel agencies and doctors). Trust in these sources facilitates adherence to vaccinations.
	Impact of emotions on vaccination decision	Emotions link vaccination to a sense of protection, reducing anxiety and increasing confidence in vaccinations.
Complacency	Perception of risk and destinations	Traveling to destinations perceived as low-risk (e.g., Europe) leads to underestimating the importance of vaccination, resulting in low motivation to vaccinate.
Convenience	Independent information search	Online research and self-information allow participants to feel more in control of their health, leading to more informed vaccination decisions.
	Experience during the COVID-19 pandemic	The pandemic highlighted the importance of easy and quick access to vaccinations but also generated more distrust towards vaccines and health information.
	Access to and clarity of information	Difficulty accessing accurate information has contributed to a distorted perception of risk, reducing the urgency to vaccinate.
Other Factors	Holistic approach to health during travel	Vaccination is viewed as part of an overall health plan, integrated with other preventive measures such as digestive medication or adapting to new environments.

## Data Availability

The datasets used and/or analyzed during the current study are available from the corresponding author on reasonable request.
